# Impacts of cost-sharing rate increment on the expenditure of outpatient care among older adults: quasi-experimental study of cost-sharing reform in Japan

**DOI:** 10.1186/s12913-026-14387-4

**Published:** 2026-03-19

**Authors:** Taro Kusama, Yudai Tamada, Manami Hoshi-Harada, Ken Osaka, Kenji Takeuchi

**Affiliations:** 1 Division of Statistics and Data Science, Liaison Center for Innovative Dentistry, Tohoku University Graduate School of Dentistry, Miyagi, Japan; 2 Department of International and Community Oral Health, Tohoku University Graduate School of Dentistry, Miyagi, Japan

**Keywords:** Health policy, Universal health insurance, Primary health care, Longitudinal studies, Health expenditures

## Abstract

**Background:**

Ensuring equitable access to healthcare under universal health coverage is critical; however, the financial sustainability of public health insurance systems faces challenges in aging societies. The present study aimed to evaluate the impact of the 2022 cost-sharing reform to increase coinsurance rate on outpatient care utilization among older adults with relatively high income, and to estimate the price elasticity of demand for outpatient care in response to the cost-sharing increase.

**Methods:**

Employing a quasi-experimental design, this cohort study treats the 2022 cost-sharing reform as a natural intervention. Data were drawn from medical claims covering individuals aged ≥75 years enrolled in the Latter-Stage Elderly Healthcare System in several Japanese prefectures. Participants included 22,013 individuals in the intervention group (coinsurance increased from 10% to 20%) and 104,461 in the control group (coinsurance remained at 10%), observed from October 1, 2021, to September 30, 2023. An increase in coinsurance rate from 10% to 20% implemented on October 1, 2022, was treated as an exposure variable. Monthly total expenditures on outpatient care—physician visits, prescriptions, and dental visits—served as primary outcomes. Changes in total expenditure post-reform were assessed using controlled interrupted time series analysis, fitting a log-gamma regression model. Relative ratios (RRs) and 95% confidence intervals were estimated, and price elasticities were calculated using the change in out-of-pocket expenditures as denominators.

**Results:**

Average total expenditures for overall outpatient care among the intervention group pre- and post-reform were ¥31,468 (SD=¥53,072) and ¥31,607 (SD=¥57,897) per month, respectively. Following the reform, expenditures significantly decreased for physician visits (RR=0.92 [0.90–0.94]) and prescriptions (RR=0.95 [0.93–0.97]) immediately after the reform, while dental visit expenditures did not significantly change (RR=0.97 [0.93–1.01]). Calculated price elasticities were highly inelastic (−0.137, −0.079, and −0.047 for physician visits, prescriptions, and dental visits, respectively).

**Conclusions:**

These findings suggest that increasing the coinsurance rate led to modest short-term reductions in outpatient care expenditures among older adults with relatively high incomes. Outpatient care demand was largely inelastic to price changes, supporting the potential for income-based cost-sharing policies to enhance the financial sustainability of public health insurance systems with limited reduction in access.

**Supplementary Information:**

The online version contains supplementary material available at 10.1186/s12913-026-14387-4.

## Introduction

In 2015, the United Nations established the Sustainable Development Goals (SDGs), with SDG 3 aiming to achieve universal health coverage (UHC) and equitable access to healthcare services for all individuals [1]. The cornerstone of UHC is the provision of a pre-payment-based public health insurance system [2]. Previous experimental interventions have demonstrated that reducing cost-sharing enhances medical care utilization and financial security [3]. However, while ensuring equitable access to healthcare is crucial in public health [4], the financial sustainability of public health insurance systems is increasingly challenged by the global demographic shift toward aging populations [5].

Japan is recognized for achieving one of the highest indices of effective health service coverage worldwide [6]. Almost all Japanese citizens are covered by the public health insurance system, which includes medical, dental, and pharmaceutical services [7]. Beneficiaries have the flexibility to choose from a wide range of authorized medical institutions, with coinsurance rates set at ≤ 30%. For older adults aged ≥ 75, except for those with high incomes (equivalent to working-age income levels), the coinsurance rate was set at 10%. However, Japan’s total health expenditures accounted for 11.4% of the gross domestic product in 2022 [8], and this figure is expected to rise steadily in the coming decades due to demographic aging [9]. In Japan’s public health insurance system, social insurance premiums are collected from all citizens according to their income [10]. Consequently, the growth in healthcare expenditures associated with population aging leads to a heavier premium burden on the working-age population. Ensuring the sustainability of the health insurance system, therefore, requires a financing scheme that promotes fairness both between and within generations.

In response to these challenges, the Japanese government implemented a policy on October 1, 2022, increasing the coinsurance rate from 10% to 20% for older adults aged ≥ 75 with relatively high incomes. This adjustment sought to address the financial disparity in medical care contributions between the older and younger generations [10]. The reform was approved by the Cabinet on December 15, 2020, and was implemented within a relatively short period [10]. Because this change in cost-sharing rates was determined at the national level and was not influenced by individual behavior, it can be regarded as an exogenous policy intervention. Accordingly, the reform provides a valuable natural experiment for estimating the causal impact of cost-sharing on outpatient care utilization [11].

Previous studies in Japan examining changes in coinsurance rates have primarily focused on reductions due to age, particularly in individuals aged over 70 [12–17]. These studies reported an increase in outpatient care utilization following reductions in the coinsurance rate, with a limited impact on inpatient care utilization [12–17]. Conversely, the 2022 reform poses a unique opportunity to explore potential shifts in medical care utilization patterns among older adults in response to increased cost-sharing. Therefore, this study aims to (1) evaluate the impact of the 2022 cost-sharing reform on outpatient care utilization among older adults aged ≥ 75 and (2) estimate the price elasticity of demand for outpatient care resulting from this reform.

## Methods

### Study design and data

This retrospective cohort study adopts a quasi-experimental design, treating the 2022 cost-sharing reform as a natural experimental intervention. The reform was implemented at a clearly defined time point, with eligibility determined by an administratively defined income threshold for adults aged 75 and over (Table 1), and applied uniformly to the eligible population [11]. Because the policy was determined at the national level and was independent of individual behavior, it can be considered exogenous. Using time-series data on healthcare expenditures and utilization among the eligible population before and after the reform allows us to contrast outcomes and estimate the causal effect of the cost-sharing reform [11].

**Table 1:** Description of the cost-sharing reform in the Latter-Stage Elderly Healthcare System in Japan

Until September 30, 2022			From October 1, 2022			Cap on the high-cost medical care benefit system	
Classification	Coinsurance rate	Population (%)^a^	Classification	Coinsurence rate	Population (%)^b^	Outpatient only (per individual)	Sum of outpatient and inpatient (per household)
Earning full salaries (Taxable income ≥ 1,450,000 JPY)	30%	1,312,518 (7.0%)	Earning full salaries (Taxable income ≥ 1,450,000 JPY)	30%	1,374,976 (7.1%)	Depending on income: 80,100–252,600 + (Total expenditure – 267,000–842,000) ×1% JPY/month^c^	
General (Taxable income > 0 JPY)	10%	9,594,719 (51.1%)	General Ⅱ (Taxable income ≥ 280,000 JPY)^d^	20%	3,853,451 (19.8%)	18,000 JPY/month^e^ [144,000 JPY/year]	57,600 JPY/month^f^
			General Ⅰ (Taxable income < 280,000 JPY)	10%	6,032,891 (31.0%)	18,000 JPY/month [144,000 JPY/year]	
Low income Ⅱ (No taxable income and total income > 80,0000 JPY)		4,829,183 (25.7%)	Low income Ⅱ (No taxable income and total income > 80,0000 JPY)		5,103,853 (26.2%)	8,000 JPY/month	24,600 JPY/month
Low income Ⅰ (No taxable income and total income ≤ 80,0000 JPY)		3,051,657 (16.2%)	Low income Ⅰ (No taxable income and total income ≤ 80,0000 JPY)		3,078,745 (15.8%)		15,000 JPY/month

^a^ Statistics were obtained from a survey of insured individuals in the Latter-Stage Elderly Healthcare System in 2022

^b^ The statistics are obtained from a survey of the insured individuals in the Latter-Stage Elderly Healthcare System in 2023

^c^ If the monthly payment at the same medical institution exceeds the cap on the high-cost medical care benefit system ≥ 3 times a year, the cap is 44,400–140,100 JPY/month

^d^ If the total income is < 2,000,000 JPY (single-person household) or < 3,200,000 JPY (≥ two-person household), “General Ⅰ (10% coinsurance rate)” is applied

^e^ Monthly out-of-pocket expenditure for outpatient care was limited to 3,000 JPY in addition to out-of-pocket expenditure after applying a 10% coinsurance rate until September 30, 2025

^f^ If the monthly payment at the same medical institution exceeds the cap on the high-cost medical care benefit system ≥ 3 times a year, the cap is 44,400 JPY/month

Data were obtained from the DeSC database (DeSC Healthcare, Inc., Tokyo, Japan), which includes individual-level medical claim information from the Latter-Stage Elderly Healthcare System (LEHS) in several prefectures across Japan. The LEHS covers almost all citizens aged ≥ 75 years in Japan. Although the availability of healthcare resources may vary across residential areas, insured individuals face essentially the same conditions for accessing care, with the only substantial variation arising from differences in cost-sharing according to income [7]. The DeSC database comprises data from approximately 12,500,000 individuals covered by health insurance societies in Japan [18], and its representativeness has been confirmed in a previous report [19].

The cost-sharing reform in the LEHS was implemented on October 1, 2022. The details of the cost-sharing reform in the LEHS are provided in Table 1. With this reform, the cost-sharing rate of older adults with relatively high incomes increased from 10% to 20%. The study period spanned 1 year before and after the cost-sharing reform, specifically from October 1, 2021, to September 30, 2023. The eligibility criteria for participants were as follows: (1) those covered by LEHS during the study period, (2) those with available income class information, and (3) those whose income class was consistently classified as “General” during October 1, 2021 − September 30, 2022 and “General I or II” during October 1, 2022 − September 30, 2023. Participants were divided into an intervention group, whose coinsurance rate increased to 20% from October 1, 2022, and a control group, whose coinsurance rate remained at 10% throughout October 1, 2021, to September 30, 2023.

Although COVID-19 cases were still being reported in Japan during the study period (October 1, 2021–September 30, 2023), no specific restrictions on outpatient care utilization were implemented by the Japanese government during this period. Previous studies have shown that outpatient utilization had largely recovered to pre-pandemic levels [20, 21]. Therefore, the influence of COVID-19 on outpatient care during our study period is likely to have been limited.

### Outpatient care expenditure and utilization

Information on outpatient care expenditure was obtained from individual receipt data generated monthly by each utilized medical facility for reimbursement purposes. “Outpatient care” was defined as medical care excluding inpatient care; therefore, home-visit medical care was considered outpatient care in this study. The total expenditure on physician visits, prescriptions, and dental visits in outpatient care was summed for each individual’s monthly data, and the overall total expenditure was calculated by summing these amounts. Overall out-of-pocket (OOP) expenditure was determined by applying the coinsurance rate and cap of the High-Cost Medical Care Benefit System to the total outpatient expenditure. The Japanese government implemented an interim measure to limit the monthly OOP expenditure for outpatient care to 3,000 JPY to minimize the impact of the cost-sharing reform from October 1, 2022, in addition to OOP expenditure after applying the 10% rate, effective until September 30, 2025 (Additional File 1: Figure S1). Subsequently, the actual coinsurance rates for individuals were recalculated and applied to the total expenditure for physician visits, prescriptions, and dental visits to compute the OOP expenditure.

Additionally, the monthly counts of utilization for physician visits, prescriptions, and dental visits were used as supplementary outcomes. Because Japan employs a free-access healthcare system, patients can adjust the frequency of their visits, whereas the intensity of care provided at each visit is largely determined by providers. Therefore, expenditures may be more suitable for capturing the actual quantity of healthcare services.

### Statistical analysis

This study employed controlled interrupted time-series analysis (ITSA) to evaluate the impact of the cost-sharing reform on outpatient care expenditures and utilization. Because ITSA relies on comparisons over time within the same population, confounding by time-invariant characteristics, including unmeasured baseline clinical and health-status factors, is likely to be minimized. Only age and sex were therefore included as covariates in our models. While single-arm ITSA was conducted only for the intervention group, the controlled ITSA incorporated both the intervention and control groups in the model, helping to account for secular trends and contemporaneous events unrelated to the reform, including time-varying factors that evolve similarly across groups [22]. Furthermore, we conducted a single-arm ITSA to investigate the robustness of the results due to changes in the control group during the intervention period. The controlled ITSA model uses the following equation [23]:

$$\begin{aligned}Y_{it}&=\alpha+\beta_1INT_{it}+\beta_2TIME_{it}+\beta_3INT_{it}\cr&\quad\cdot TIME_{it}+\beta_4CTRL_{it}+\beta_5TIME_{it}\cr&\quad\cdot CTRL_{i}+\beta_6AGE1_{it}+\beta_7AGE2_{it}\cr&\quad+\beta_8SEX_{it}+\varepsilon_{it}\end{aligned}$$

Where Y_it_ is the outcome measure for individual i at time t. Similarly, TIME is the time variable based on October 1, 2021, INT is the dummy variable set to 0 before October 1, 2022, and 1 after October 1, 2022, for the intervention group, and CTRL is the dummy variable set to 0 for the intervention group and 1 for the control group. AGE1, AGE2, and SEX are dummy variables for age groups (80–84 or 85 + years) and sex (female = 1, male = 0), respectively. β1​, β2​, and β3​ represent the changes due to the increase in coinsurance rate to 20%, the trend before the increase, and the slope change after the increase, respectively.

We applied generalized estimating equations (GEE) using a gamma distribution with a log-link function. Relative ratios (RRs) and 95% confidence intervals (CIs) were estimated using sandwich standard error estimators. Moreover, a two-part model for expenditure was employed in the sensitivity analysis due to the high number of zero expenditures in the monthly data [24]. Logistic and log-gamma regression models were fitted to the first and second parts of the model, respectively. To address the short-term effects immediately before and after the cost-sharing reform, a donut-hole analysis was performed, excluding the 2 months before and after the intervention (August 1, 2022–November 30, 2022) as a sensitivity analysis based on previous studies [12]. For the analysis employing the monthly counts of outpatient care utilization as outcome, GEE using a negative binomial distribution with a log-link function was fitted.

To assess the impact of the increase in OOP expenditure on the demand for outpatient care, we calculated both the point and arc price elasticity of demand [25], using the following equations:

$$\begin{aligned}&\text{Point price elasticity}=\frac{\varDelta Q/{Q}_{1}}{\varDelta P/{P}_{1}},\cr& \text{Arc price elasticity}=\frac{{P}_{1}+{P}_{2}}{{Q}_{1}+{Q}_{2}}\times\frac{\varDelta Q}{\varDelta P}\end{aligned}$$

Where Q_1_ and Q_2_ are total expenditures before and after the increase in the coinsurance rate to 20%, P_1_ and P_2_ are OOP expenditures before and after the increase, and ΔQ and ΔP represent the changes in expenditures and prices, respectively. Each estimate is standardized using a parametric g-formula based on a regression model of the controlled ITSA. All statistical analyses were performed using Stata/MP software (version 17.0; Stata Corp.). Statistical significance level was set at *α* = 0.05. We adhered to the STROBE guidelines for reporting our findings.

### Ethical issues

For this study, anonymized information obtained from the commercially available DeSC Database (DeSC Healthcare, Inc., Tokyo, Japan) was used. The anonymization procedure adhered to the Next-Generation Medical Infrastructure Act and the Japan Act on the Protection of Personal Information. Based on these acts, informed consent was waived due to the anonymous nature of the data. According to the ethical guidelines for clinical research in Japan, studies using anonymized processed information do not require review by an ethics review committee. This study was conducted in accordance with the principles of the Declaration of Helsinki and ethical guidelines for clinical research in Japan.

## Results

### Sample characteristics

A total of 22,013 and 104,461 participants were included in the intervention and control groups, respectively, representing 3,035,376 person-month observations (Additional File 1: Figure S2). Descriptive statistics of the participants are provided in Additional File 1: Table S1.

### Trends in outpatient care expenditures

**Fig. 1:**
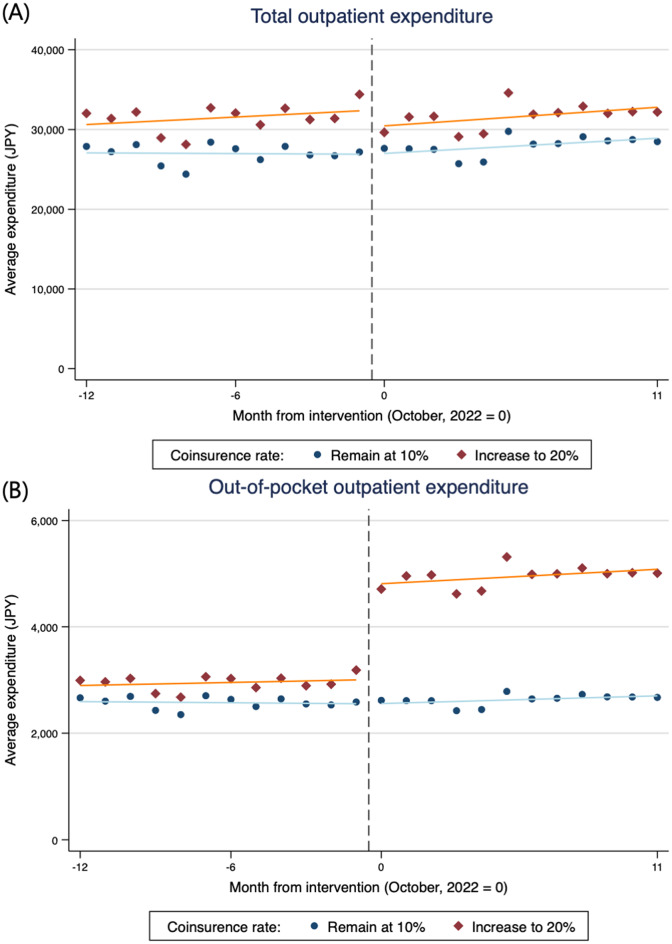
(**A**) Total and (**B**) out-of-pocket expenditure of overall outpatient care per person-month (*n* = 126,474). NOTE 1: Each plot represents the monthly average expenditure per group. The solid line is fitted separately using ordinary least squares for the periods before and after the intervention. NOTE 2: Out-of-pocket outpatient care expenditure was calculated by applying the coinsurance rate and high-cost medical expense benefits to the total outpatient expenditure

**Fig. 2:**
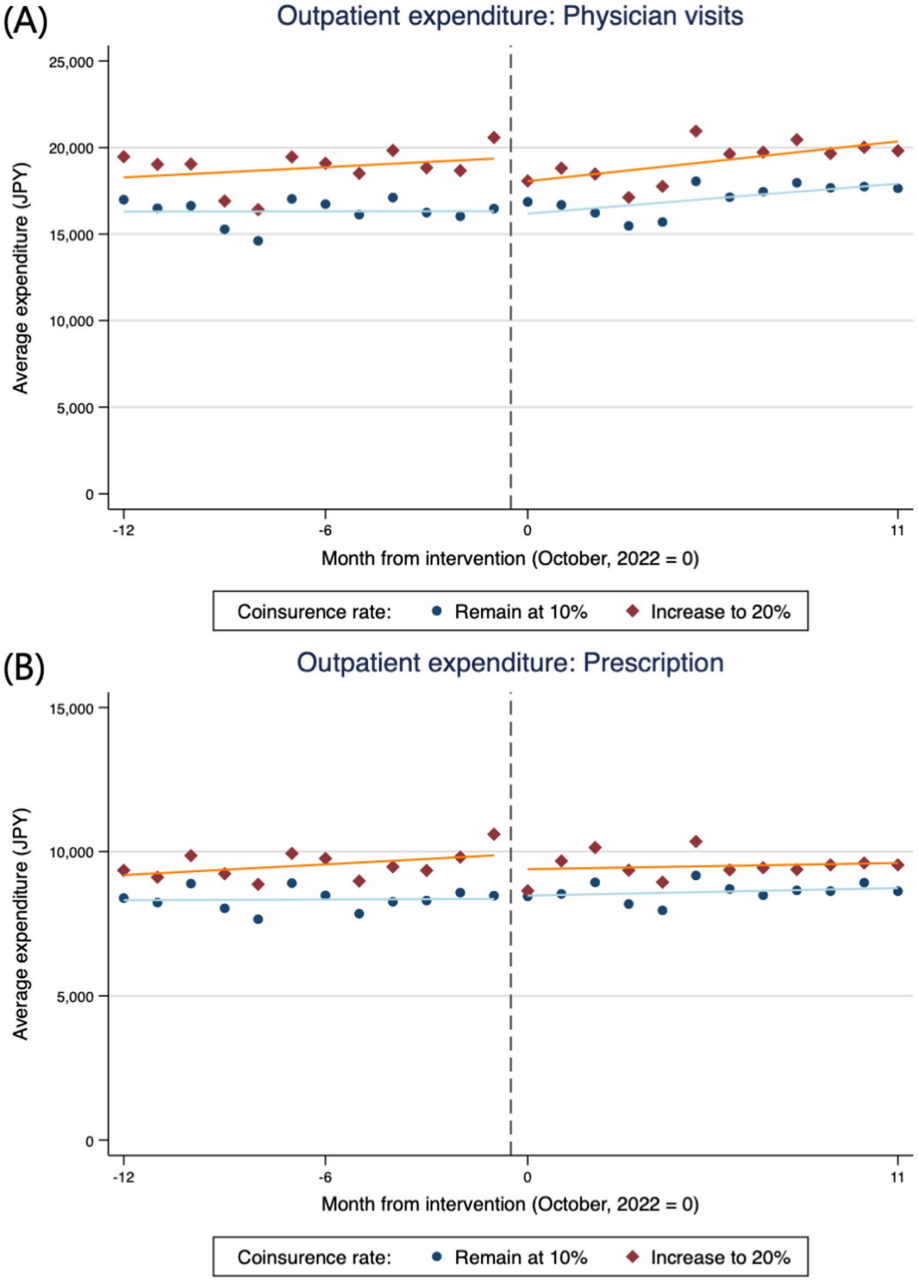

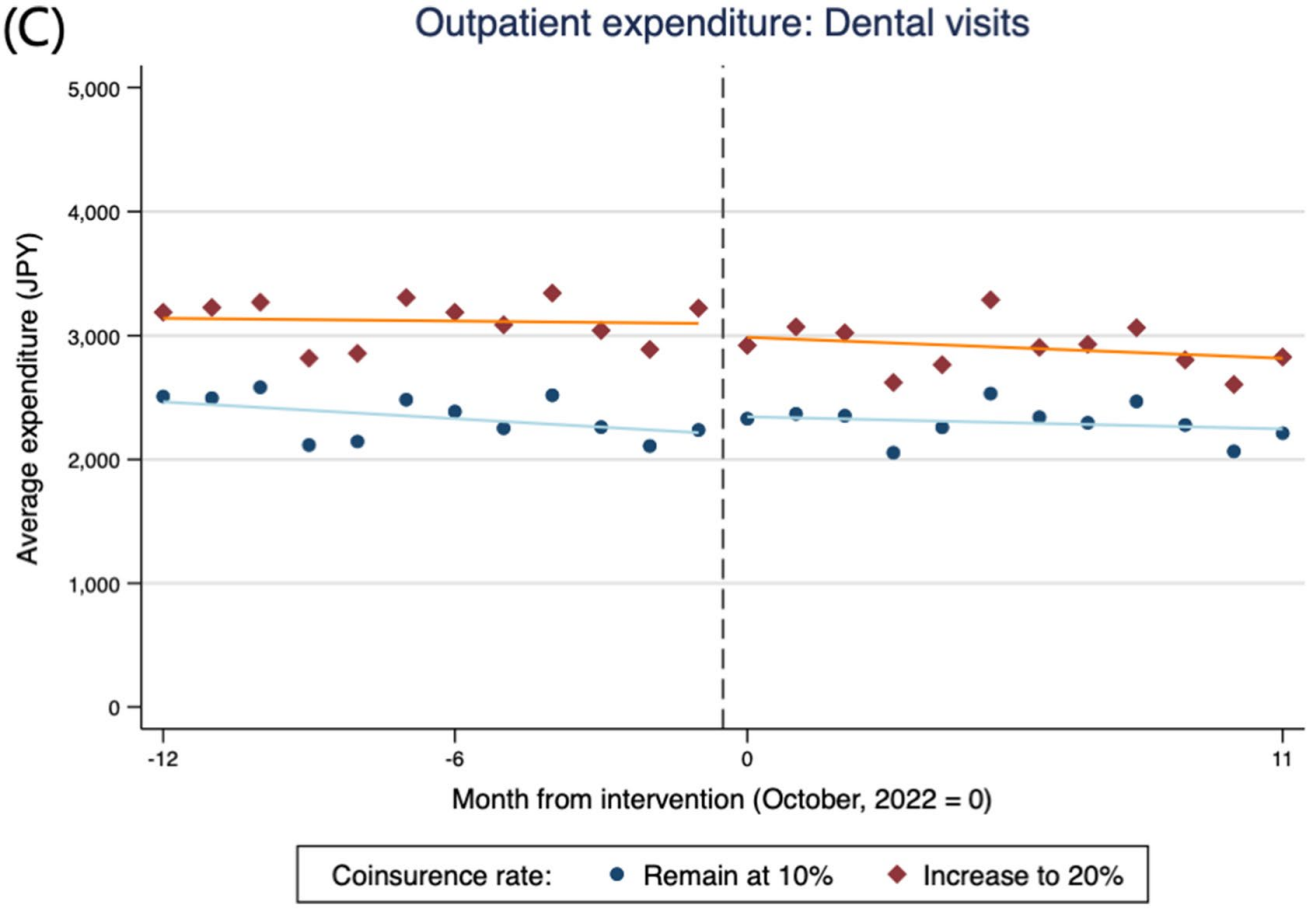
Total expenditure of outpatient care per person-month by (**A**) physician visits, (**B**) prescription, and (**C**) dental visits (*n* = 126,474). NOTE: Each plot represents the monthly average expenditure per group. The solid line is fitted separately using ordinary least squares for the periods before and after the intervention

Figure 1 shows plots of the average monthly total and OOP expenditures for outpatient care. A decrease in total outpatient care expenditure is graphically observed only in the intervention group before and after the cost-sharing reform on October 1, 2022. Although a small decline in total expenditure was graphically observed after the reform, it gradually recovered to its previous level. Conversely, OOP expenditures increased following the cost-sharing reform in the intervention group. Figure 2 shows plots of the average monthly total outpatient care expenditure for physician visits, prescriptions, and dental visits. For all types of outpatient care, a small decrease in total expenditure was observed. Similar trends were also observed in the plots based on outpatient care utilization counts (Additional File 1: Figure S3).

### Controlled interrupted time series analysis

As shown in Table 2, the increased cost-sharing was associated with a significant decrease in total expenditure for overall outpatient care (RR = 0.93 [95% CI = 0.92, 0.95]). Significant decreases were observed in the total expenditure on physician visits (RR = 0.92 [95% CI = 0.90, 0.94]) and prescriptions (RR = 0.95 [95% CI = 0.93, 0.97]); however, no significant decrease in outpatient care expenditure on dental visits was observed (RR = 0.97 [95% CI = 0.93, 1.01]).

**Table 2:** Association between the increase in coinsurance rate and outpatient care expenditure based on the controlled interrupted time series analysis (*n* = 126,474)

Total expenditure	Whole period model		Donut-hole model^a^	
	RR (95% CI)	*P*-value	RR (95% CI)	*P*-value
Overall outpatient care				
Change due to the increase in CIR to 20%	0.93 (0.92, 0.95)	< 0.001	0.96 (0.94, 0.98)	0.001
Trend before the increase in CIR to 20%	1.005 (1.003, 1.007)	< 0.001	1.001 (0.999, 1.004)	0.174
Slope change after the increase in CIR to 20%	1.002 (0.999, 1.005)	0.157	1.005 (1.002, 1.009)	0.002
Physician visits				
Change due to the increase in CIR to 20%	0.92 (0.90, 0.94)	< 0.001	0.93 (0.89, 0.96)	< 0.001
Trend before the increase in CIR to 20%	1.005 (1.003, 1.008)	< 0.001	1.002 (0.999, 1.005)	0.169
Slope change after the increase in CIR to 20%	1.006 (1.002, 1.020)	0.003	1.011 (1.006, 1.016)	< 0.001
Prescription				
Change due to the increase in CIR to 20%	0.95 (0.93, 0.97)	< 0.001	1.03 (1.00, 1.07)	0.060
Trend before the increase in CIR to 20%	1.006 (1.004, 1.009)	< 0.001	1.000 (0.998, 1.003)	0.817
Slope change after the increase in CIR to 20%	0.996 (0.992, 0.999)	0.022	0.997 (0.993, 1.002)	0.301
Dental visits				
Change due to the increase in CIR to 20%	0.97 (0.93, 1.01)	0.134	0.95 (0.90, 1.00)	0.067
Trend before the increase in CIR to 20%	0.998 (0.994, 1.002)	0.338	1.000 (0.994, 1.005)	0.862
Slope change after the increase in CIR to 20%	0.996 (0.990, 1.003)	0.259	0.996 (0.988, 1.003)	0.291
Out-of-pocket expenditure: Overall outpatient care	RR (95% CI)	*P*-value	RR (95% CI)	*P*-value
Change due to the increase in CIR to 20%	1.60 (1.58, 1.61)	< 0.001	1.64 (1.61, 1.66)	< 0.001
Trend before the increase in CIR to 20%	1.003 (1.002, 1.004)	< 0.001	1.000 (0.999, 1.001)	0.966
Slope change after the increase in CIR to 20%	1.002 (1.000, 1.003)	0.040	1.005 (1.003, 1.007)	< 0.001

^a^ Donut-hole model excluded data before and after 2 months of intervention (August 8, 2022, to November 30, 2022)

NOTE: The estimates were predicted using standardization based on the regression model as follows: Y_it_ = α + β_1_INT_it_ + β_2_TIME_it_ + β_3_INT_it_・TIME_it_ + β_4_CTRL_it_ + β_5_TIME_it_・CTRL_it_ + β_6_AGE1_it_ + β_7_AGE2_it_ + β_8_SEX_it_ + ε_it_ where Y: outcome variable, INT: intervention to 20% coinsurance rate from 10% TIME: baseline per-month trend, CTRL: treatment/control groups indicator, AGE1: age group of 80–84 years, AGE2: age group of 85+ years, and SEX: sex

Abbreviations: RR, relative ratio; 95% CI, 95% confidence interval; CIR, coinsurance rate

In the donut-hole model, excluding the 2 months before and after the reform, although a significant decrease in total expenditure for overall outpatient care and physician visits was noted, the magnitude of the decrease became smaller than that of the whole-period models. Furthermore, no significant decrease in the total expenditure for prescriptions or dental visits was observed. For physician visits, the slope of the trend significantly increased in both the whole period and the donut-hole model (*P* < 0.01), indicating that the decrease in total expenditure due to the increase in the coinsurance rate gradually recovered to its previous level.

Contrarily, OOP expenditure significantly increased with the increase in the coinsurance rate by 1.60 (95% CI = 1.58, 1.61) and 1.64 (95% CI = 1.61, 1.66) in the whole period and donut hole models, respectively.

The results of the controlled ITSA using outpatient care utilization counts as the outcome variable are presented in Additional File 1, Table S2, and they show patterns similar to those observed for total expenditures.

### Sensitivity analyses

The results of the single-arm ITSA were consistent with those of the controlled ITSA (Additional File 1: Table S3). The estimates from the two-part model are presented in Additional File 1: Table S4. The first-part model estimates suggested that utilization of physician visits and prescriptions significantly decreased with an increase in the coinsurance rate (*P* < 0.001), whereas the second-part model indicated that the total expenditure among those who utilized outpatient care decreased significantly only for physician visits (*P* < 0.001).

### Price elasticity estimates

Price elasticity, calculated using estimates based on the controlled ITSA, is provided in Table 3. The point and arc price elasticities for outpatient care were − 0.109 and − 0.146 for overall outpatient care, − 0.137 and − 0.182 for physician visits, − 0.079 and − 0.108 for prescriptions, and − 0.047 and − 0.063 for dental visits. However, the change in total expenditure on dental visits was not significant (*P* > 0.05), and the price elasticity for dental visits could be zero. Similar price elasticity was obtained using the two-part model (Additional File 1: Table S5). For the price elasticity obtained from the donut hole model, similar values of price elasticity were observed for overall outpatient care, physician visits, and dental visits; however, the price elasticity for prescriptions was positive and inelastic (Additional File 1: Table S6). However, the change in total expenditure on prescriptions was not significant (*P* > 0.05), and the price elasticity for prescriptions could be zero. Collectively, all the calculated values of price elasticity were close to zero, indicating that outpatient care expenditure is highly inelastic to an increase in price. Price elasticity calculated from the change in outpatient care utilization counts also indicated highly inelastic responses to the increase in the coinsurance rate (Additional File 1: Table S7).

**Table 3:** Price elasticity of outpatient care expenditure in response to an increase in coinsurance rate (n = 22,013)

	Outpatient care expenditure											
	Overall outpatient care			Physician visits			Prescription			Dental visits		
	Mean^a^ (JPY)	95% CI		Mean^a^ (JPY)	95% CI		Mean^a^ (JPY)	95% CI		Mean^a^ (JPY)	95% CI	
		LL	UL		LL	UL		LL	UL		LL	UL
Total expenditure												
Q_1_: Before the increase in CIR to 20%	32,627	32,077	33,178	19,808	19,389	20,227	9,762	9,465	10,058	3,085	3,006	3,164
Q_2_: After the increase in CIR to 20%	30,506	29,989	31,022	18,288	17,879	18,697	9,254	8,998	9,510	2,994	2,916	3,071
∆Q = Q_2_ − Q_1_	−2,122	−2,594	−1,649	−1,520	−1,926	−1,113	−508	−705	−310	−91	−210	28
Out-of-pocket expenditure												
P_1_: Before the increase in CIR to 20%	3,023	2,989	3,056	1,827	1,801	1,852	894	878	909	305	298	313
P_2_: After the increase in CIR to 20%	4,823	4,764	4,883	2,849	2,802	2,896	1,482	1,451	1,513	496	485	508
∆P = P_2_ − P_1_	1,801	1,749	1,852	1,022	979	1,066	589	564	613	191	177	206
Point price elasticity	−0.109	-		−0.137	-		−0.079	-		−0.047	-	
Arc price elasticity	−0.146	-		−0.182	-		−0.108	-		−0.063	-	

NOTE 1: The estimates were predicted using standardization based on the regression model as follows: Y_it_ = α + β_1_INT_it_ + β_2_TIME_it_ + β_3_INT_it_・TIME_it_ + β_4_CTRL_it_ + β_5_TIME_it_・CTRL_it_ + β_6_AGE1_it_ + β_7_AGE2_it_ + β_8_SEX_it_ + ε_it_ where Y: outcome variable, INT: intervention to 20% coinsurance rate from 10% TIME: baseline per-month trend, CTRL: treatment/control groups indicator, AGE1: age group of 80–84 years, AGE2: age group of 85 + years, and SEX: sex

NOTE 2: Point price elasticity =$\frac{\varDelta Q/{Q}_{1}}{\varDelta P/{P}_{1}}$, Arc price elasticity =$\frac{{P}_{1}+{P}_{2}}{{Q}_{1}+{Q}_{2}}\times\frac{\varDelta Q}{\varDelta P}$

Abbreviations: 95% CI, 95% confidence interval; LL, lower limit; UL, upper limit; CIR, coinsurance rate

## Discussion

### Summary of the key findings

This study evaluated the impact of an increase in the coinsurance rate from 10% to 20% due to the cost-sharing reform in Japan on outpatient care expenditures among older adults with relatively high incomes. The results demonstrated a significant decrease in total expenditure for overall outpatient care, physician visits, and prescriptions due to the increased coinsurance rate. In contrast, the total expenditure on dental visits did not change significantly. However, the total expenditure for overall outpatient care and physician visits gradually increased after the reform, and the difference in the total expenditure for prescriptions was not significant in the donut hole model. Moreover, the calculated price elasticity for the total expenditure on outpatient care following an increase in the coinsurance rate was small. These results suggest that although the total expenditure on outpatient care decreased significantly immediately after the coinsurance rate increased, the impact on outpatient care utilization was limited to a temporary period after the intervention, and its long-term impact may be limited.

### Comparison with previous findings

These findings were consistent with those of previous studies. Most earlier studies investigating the impact of changes in cost-sharing on medical utilization among older Japanese adults have mainly focused on reductions in the coinsurance rate at the age of 70 [12–17]. While previous studies evaluated the opposite direction of change in the coinsurance rate compared to the present study, they similarly reported a significant increase in outpatient care utilization when the coinsurance rate was reduced [12–17]. Moreover, two previous studies reported price elasticities for outpatient care of − 0.07 and − 0.16 following reductions in the coinsurance rate from 30% to 10% at age 70 in Japan, values that are similar to those obtained in the present study [12, 15]. To the best of our knowledge, no previous study has evaluated the impact of increases in coinsurance rates among adults aged ≥ 75 in Japan following the 2022 reform.

Our study adds to the existing literature by showing that increases in coinsurance rates also influence outpatient care utilization, with price elasticities that are nearly symmetric with respect to the direction of the coinsurance change, although they remain highly inelastic. Although the 2022 reform applied only to adults aged ≥ 75 with relatively high income, the elasticities observed in our study were consistent with estimates from earlier reforms that were not income-restricted [12, 15]. These findings suggest that the inelastic response to changes in coinsurance may be relatively stable across age and income groups, particularly among older adults. Nevertheless, we acknowledge that direct evidence among low-income and younger populations remains limited and warrants further investigation.

In this study, no statistically significant decrease in outpatient care expenditure for dental visits was observed following the cost-sharing reform. While a previous study using a self-reported questionnaire reported an increase in dental visits after a reduction in coinsurance rates [17], this study provides the first claims-based evidence in Japan, suggesting that changes in coinsurance rates may have a limited effect on dental care utilization.

Price elasticity is influenced by both the substitution effect and the income effect [25]. In terms of substitution, there are few alternatives for medical care services, leading to low price elasticity. Regarding the income effect, the proportion of income spent on medical care also affects price elasticity: a higher proportion of income spent on specific goods leads to higher price elasticity. In this study, the average OOP expenditure for dental visits was lower than that for physician visits and prescriptions, accounting for only 10% of the overall outpatient care expenditure, which may explain the minimal change in dental care expenditure and lower price elasticity. Furthermore, previous studies have indicated that dental care utilization is strongly influenced by socioeconomic status. The 2022 cost-sharing reform primarily targeted higher-income older adults aged ≥ 75, specifically those in the top 7–27 percentiles of income; therefore, the impact of the increased OOP expenditure on dental visits may have been limited.

During the study period, the Japanese government implemented an interim measure that limited the monthly OOP expenditure for outpatient care to an additional 3,000 yen above the 10% coinsurance rate. This may have attenuated the observed increase in OOP expenditures. Nevertheless, the average increase in OOP expenditures in our data was approximately 1,800 JPY (Table 3), which is clearly below this temporary monthly cap. In addition, the proportion of individuals whose expenditures were subject to the High-Cost Medical Care Benefit System increased only slightly during the study period (Additional File 1: Figure S4). Therefore, even in the absence of this interim cap, the overall impact on outpatient expenditures and utilization would likely have been limited.

### Implications

This study has several important implications for cost-sharing policies. The reform resulted in a short-term decline in total outpatient care expenditures but showed no sustained reduction over time. This pattern suggests that older adults with relatively high incomes may temporarily adjust their utilization in response to higher coinsurance, yet they do not appear to forego necessary outpatient care in the long run. In this sense, the reform is unlikely to have generated a persistent impact on access to care within this population.

At the same time, the reform increased OOP payments only for older adults with relatively high incomes, representing a rare example of an income-differentiated coinsurance policy. From the perspective of financing equity, such a design strengthens the principle of vertical equity—those with greater ability to pay contribute relatively more—while preserving financial protection for lower-income older adults, whose coinsurance rate remained unchanged. Moreover, premiums in Japan’s public health insurance system are already determined according to individual or household income and collected from all insured, including older adults. In addition to this income-based premium structure, the current system also differentiates cost-sharing by income and age for older adults. The normative justification for maintaining these dual income-based mechanisms of financing deserves further consideration in future policy discussions.

The financial burden of medical expenditure for older adults is a critical challenge in aging societies [5]. While UHC is essential for reducing health disparities [6], ensuring distributive fairness in financial contributions—both between and within generations—is equally important for the long-term sustainability of UHC [26]. In this context, the 2022 reform may contribute to system sustainability by modestly shifting the financial burden toward high-income older adults, thereby alleviating part of the contribution burden on the working-age population, without causing sustained reductions in outpatient service use. To address uncertainty in healthcare needs across generations, a fairer and more balanced financing scheme within the public health insurance system is required.

Previous studies have reported that changes in cost-sharing affect healthcare utilization, whereas the impact on health status is generally limited [27]. Although the current study did not assess health outcomes directly, prior research on cost-sharing reforms in Japan has demonstrated limited effects on health [12, 15], and similar consequences may be expected for the 2022 reform. Nevertheless, further research is warranted to assess potential longer-term health and distributional impacts.

Japan’s public health insurance system adopts a social insurance model. Although multiple insurers exist depending on age and employment status, virtually all residents receive the same benefit package based on payment of income-related social insurance premiums. The findings of this study may therefore be applicable to settings such as Korea or Taiwan [28, 29], where a single insurer administers public health insurance for the entire population. However, caution is warranted when generalizing to more complex social insurance systems such as those in Germany or France, which—despite also being based on social insurance principles—are more fragmented and institutionally diverse [30].

### Limitations and strengths

This study has certain limitations. First, the participants were drawn from several prefectures in Japan, which limited the representativeness of the targeted population. However, the study used a before-and-after comparison within individuals based on the ITSA, and the internal validity of the results was less likely to be influenced by the representativeness of the sample. Second, the observation period was limited to one year before and after the cost-sharing reform, which restricted our ability to evaluate longer-term behavioral responses. Nevertheless, because we observed only minimal changes during the first post-reform year, substantial long-term effects may be less likely; however, future studies with longer follow-up are warranted.

Despite these limitations, this study had notable strengths. First, it used a quasi-experimental design with controlled ITSA, providing results with higher internal validity and causal interpretation. Second, the study included a large sample size of > 120,000 participants, encompassing both the intervention and control groups, which enhanced the statistical power of the estimates.

## Conclusion

This quasi-experimental study evaluated the impact of the increase in the coinsurance rate from 10% to 20% on outpatient care expenditure among Japanese older adults aged ≥ 75 with relatively high incomes. The findings indicate that outpatient care expenditures for physician visits significantly decreased immediately after the cost-sharing reform, whereas changes in outpatient care expenditures for prescriptions and dental visits remain unclear. Additionally, the calculated price elasticities for outpatient care were small across physician visits, prescriptions, and dental visits, indicating that these expenditures were highly inelastic with respect to increases in OOP costs. Ensuring distributive fairness of financial contributions across and within generations is essential for sustainable public health insurance systems.

## Supplementary Information

Below is the link to the electronic supplementary material.

Supplementary Material 1

## Data Availability

The data supporting the findings of this study are available from DeSC Healthcare, Inc. However, restrictions apply to the availability of these data, which were used under the license for the current study and are not publicly available. However, the data are available from the corresponding author upon reasonable request and with permission from DeSC Healthcare, Inc. (https://desc-hc.co.jp/en).

## Abbreviations

CI: Confidence interval
ITSA: Interrupted time-series analysis
LEHS: Latter-Stage Elderly Healthcare System
OOP: Out-of-pocket
RR: Relative ratio
SD: Standard deviation
SDGs: Sustainable Development Goals
UHC: Universal health coverage
