# Supplementary material for: Impacts of cost-sharing rate increment on the expenditure of outpatient care among older adults: quasi-experimental study of cost-sharing reform in Japan
Source: BMC Health Serv Res. 2026 Mar 19;26:599. doi: 10.1186/s12913-026-14387-4 (PMC13123153; doi:10.1186/s12913-026-14387-4)
Supplement: Supplementary file 1 — Supplementary Material 1 [file 12913_2026_14387_MOESM1_ESM.docx]

Supplementary materials

**Impacts of cost-sharing rate increment on the expenditure of outpatient care among older adults: Quasi-experimental study of cost-sharing reform in Japan**

**Authors:** Taro Kusama, PhD^1,2*^; Yudai Tamada, PhD^2^; Manami Hoshi-Harada, PhD^2^; Ken Osaka, PhD^2^; Kenji Takeuchi, PhD^1,2^

**Affiliations:**

^1^ Division of Statistics and Data Science, Liaison Center for Innovative Dentistry, Tohoku University Graduate School of Dentistry, Miyagi, Japan

^2^ Department of International and Community Oral Health, Tohoku University Graduate School of Dentistry, Miyagi, Japan

***Corresponding author:**

Taro Kusama

Division of Statistics and Data Science

Liaison Center for Innovative Dentistry

Tohoku University Graduate School of Dentistry

4-1 Seiryo-machi, Aoba-ku, Sendai 980-8575, Japan

Phone: +81-22-717-7639

Email: [taro.kusama.a2@tohoku.ac.jp](mailto:taro.kusama.a2@tohoku.ac.jp)

*Table of Contents*

- Figure S1. Description of an interim measure to limit the monthly OOP expenditure for outpatient care to 3,000 JPY in addition to OOP expenditure after applying a 10% coinsurance rate until 2025/09/30, implemented by the Japanese government.
- Figure S2. Flow diagram of the participants inclusion.
- Table S1. Descriptive statistics of the participants (n = 126,474).
- Figure S3. Utilization counts of outpatient care per person-month by (A) physician visits, (B) prescription, and (C) dental visits (n = 126,474).
- Table S2. Association between the increase in coinsurance rate and outpatient care utilization based on the controlled interrupted time series analysis (n = 126,474)
- Table S3. Association between increase of coinsurance rate and outpatient care expenditure and utilization based on single arm interrupted time series analysis (n = 22,013).
- Table S4. Association between the increase of coinsurance rate and outpatient care expenditure based on the two-part model (n = 126,474).
- Table S5. Price elasticity of outpatient care expenditure due to increase of coinsurance rate based on two-part model (n = 22,013).
- Table S6. Price elasticity of outpatient care expenditure due to increase of coinsurance rate based on donut hole model (n = 22,013).
- Table S7. Price elasticity of outpatient care utilization due to increase of coinsurance rate (n = 22,013).
- Figure S4. The proportion of individuals whose expenditures were subject to the High-Cost Medical Care Benefit System (n = 22,013).

**
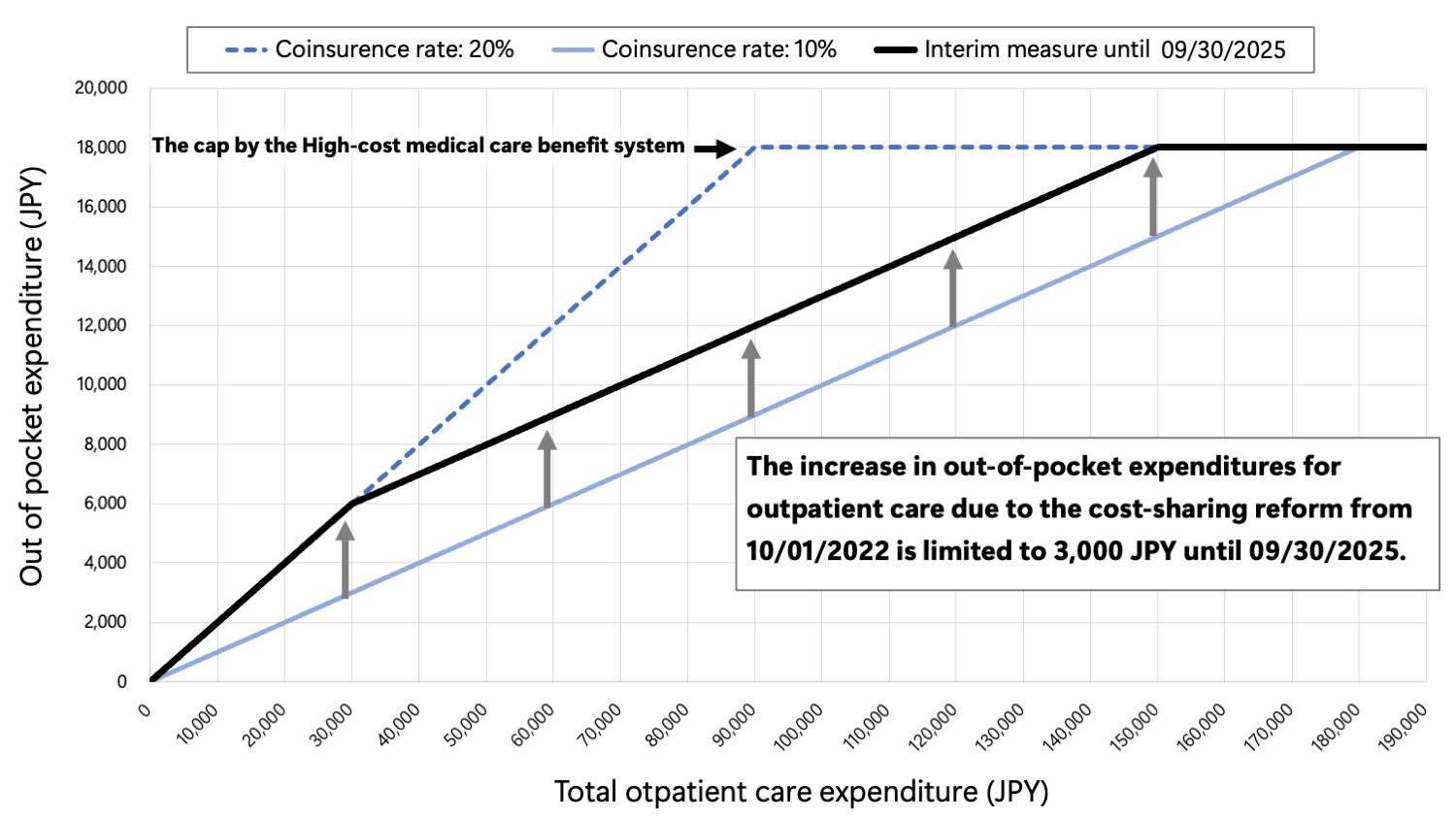
**

**Figure S1. Description of an interim measure to limit the monthly OOP expenditure for outpatient care to 3,000 JPY in addition to OOP expenditure after applying a 10% coinsurance rate until 2025/09/30, implemented by the Japanese government.**


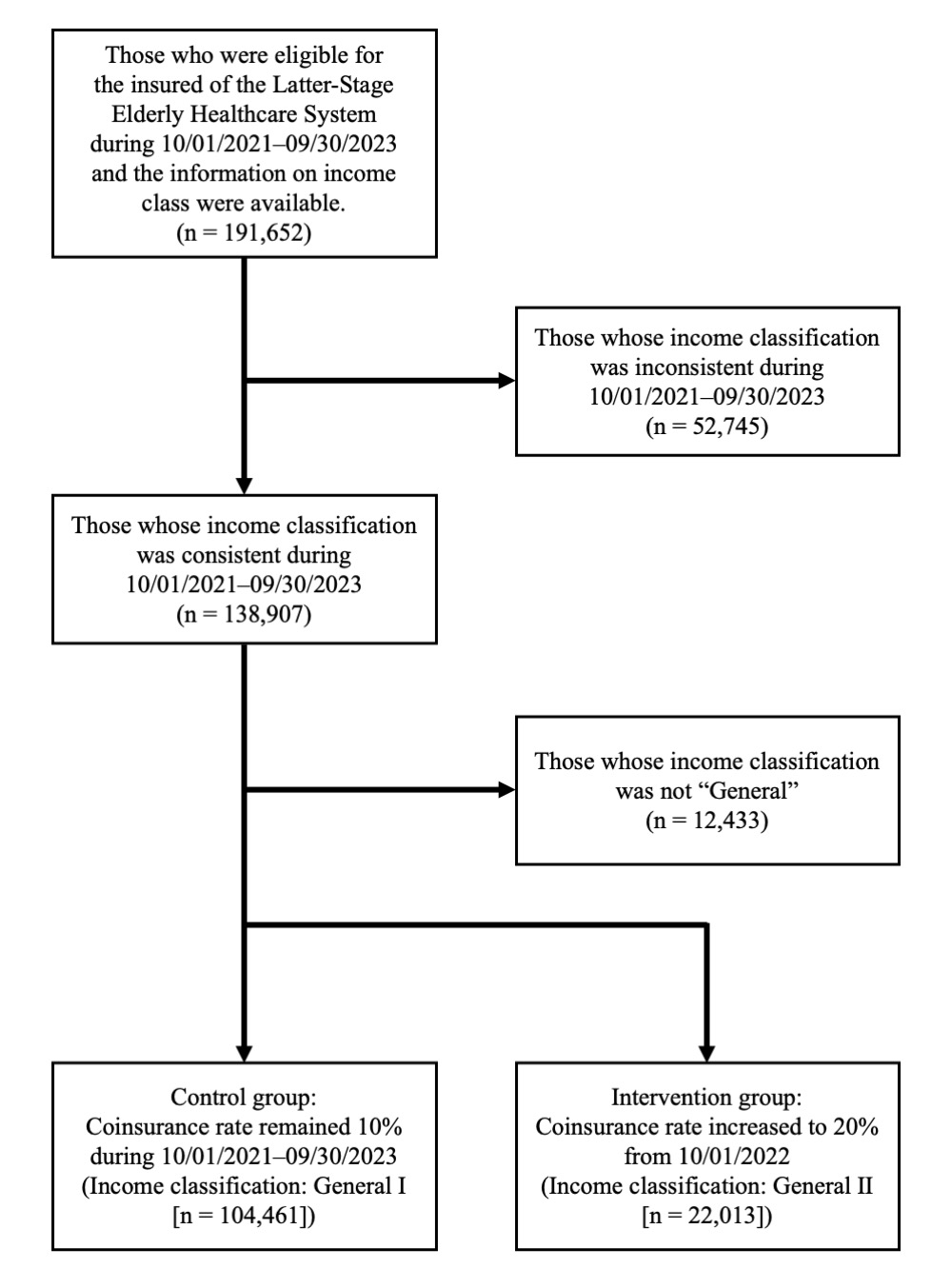


**Figure S2. Flow diagram of the participants inclusion.**

**Table S2. Descriptive statistics of the participants (n = 126,474).**

|  |  | Coinsurance rate | | | |
| --- | --- | --- | --- | --- | --- |
|  |  | Remained 10% for whole period (n = 104,461) | | Increased to 20% from 2022/10/01 (n = 22,013) | |
| Total outpatients care expenditure (JPY/month) ^a^ | | mean | SD | mean | SD |
| Overall | 10/01/2021–09/30/2022 | 26,987 | 44,338 | 31,468 | 53,072 |
|  | 10/01/2022–09/30/2023 | 27,952 | 49,071 | 31,607 | 57,897 |
| Physician visits | 10/01/2021–09/30/2022 | 16,308 | 35,202 | 18,822 | 40,882 |
|  | 10/01/2022–09/30/2023 | 17,051 | 39,115 | 19,209 | 46,459 |
| Prescription | 10/01/2021–09/30/2022 | 8,337 | 21,425 | 9,526 | 27,939 |
|  | 10/01/2022–09/30/2023 | 8,604 | 24,006 | 9,497 | 28,664 |
| Dental visits | 10/01/2021–09/30/2022 | 2,342 | 8,624 | 3,120 | 9,747 |
|  | 10/01/2022–09/30/2023 | 2,297 | 8,485 | 2,902 | 9,035 |
| Overall  out-of-pocket expenditure ^b^ | 10/01/2021–09/30/2022 | 2,574 | 2,886 | 2,948 | 3,238 |
|  | 10/01/2022–09/30/2023 | 2,629 | 2,999 | 4,947 | 6,404 |
| Baseline characteristics | | n | % | n | % |
| Sex | Male | 35,229 | 33·7 | 13,012 | 59·1 |
|  | Female | 69,232 | 66·3 | 9,001 | 40·9 |
| Age (years) | 75–79 | 39,590 | 37·9 | 8,512 | 38·7 |
|  | 80–84 | 31,883 | 30·5 | 7,054 | 32·0 |
|  | 85+ | 32,988 | 31·6 | 6,447 | 29·3 |

^a^ Means and standard deviations were calculated from the pooled data of all participants' values for each month.

^b^ After applying for High-Cost Medical Expense Benefit.


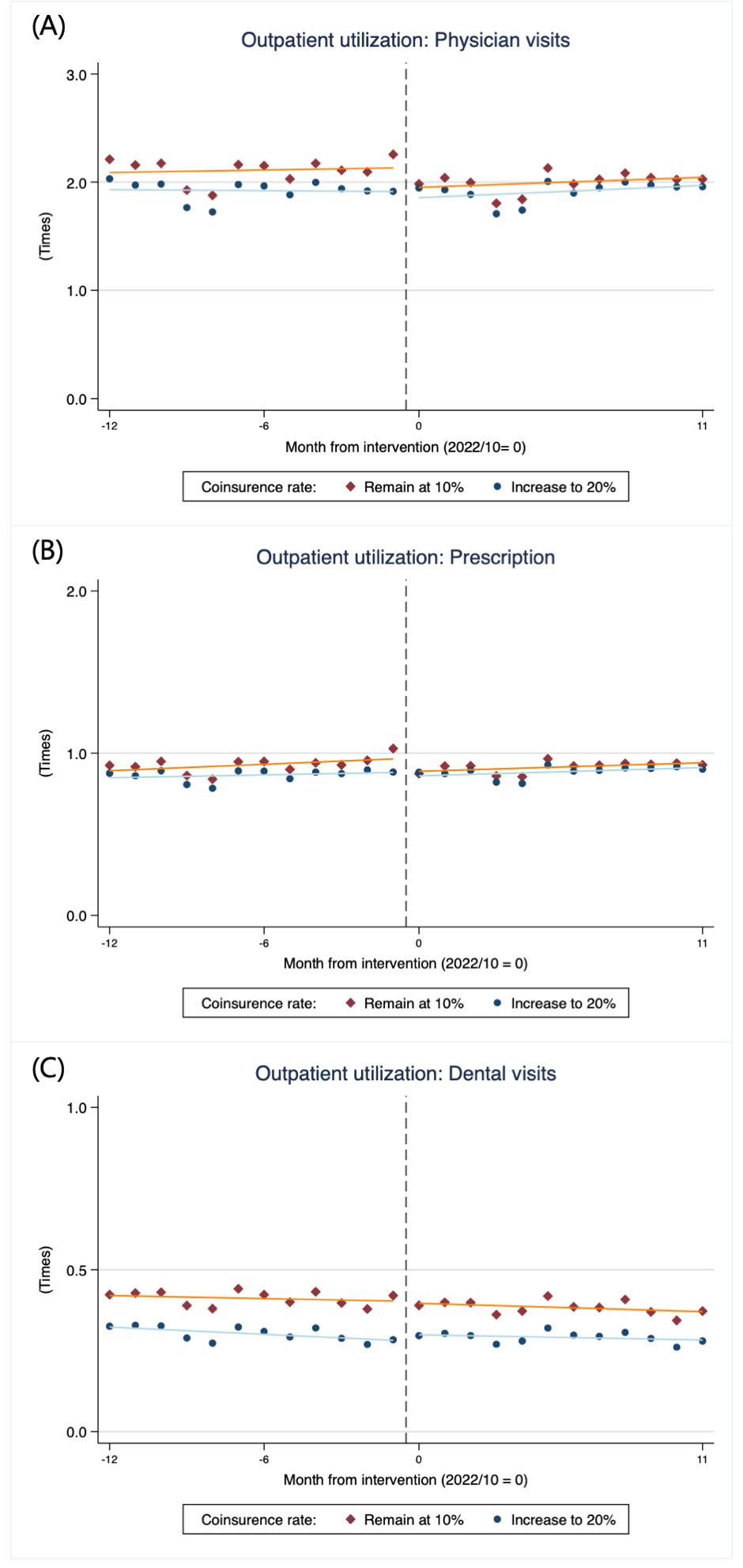


**Figure S3. Utilization counts of outpatient care per person-month by (A) physician visits, (B) prescription, and (C) dental visits (n = 126,474).**

NOTE: Each plot represents the monthly average utilization counts per group. The solid line is fitted separately using ordinary least squares for the periods before and after the intervention.

**Table S2. Association between the increase in coinsurance rate and outpatient care utilization based on the controlled interrupted time series analysis (n = 126,474)**

| Physician visits | RR | 95% CI | | p-value |
| --- | --- | --- | --- | --- |
|  |  | LL | UL |  |
| Change due to the increase to 20% coinsurance rate | 0.91 | 0.90 | 0.92 | <0.001 |
| Trend before the increase to 20% coinsurance rate (per month) | 1.002 | 1.001 | 1.003 | 0.006 |
| Slope change after the increase to 20% coinsurance rate (per month) | 1.002 | 1.000 | 1.004 | 0.024 |
| Prescription | RR | 95% CI | | p-value |
|  |  | LL | UL |  |
| Change due to the increase to 20% coinsurance rate | 0.92 | 0.91 | 0.92 | <0.001 |
| Trend before the increase to 20% coinsurance rate (per month) | 1.007 | 1.006 | 1.008 | <0.001 |
| Slope change after the increase to 20% coinsurance rate (per month) | 0.998 | 0.997 | 1.000 | 0.017 |
| Dental visits | RR | 95% CI | | p-value |
|  |  | LL | UL |  |
| Change due to the increase to 20% coinsurance rate | 0.99 | 0.96 | 1.02 | 0.462 |
| Trend before the increase to 20% coinsurance rate (per month) | 0.996 | 0.993 | 0.999 | 0.007 |
| Slope change after the increase to 20% coinsurance rate (per month) | 0.998 | 0.994 | 1.003 | 0.403 |

NOTE: The estimates were predicted using standardization based on the regression model as follows: *Y_it_ = α + β_1_INT_it_ + β_2_TIME_it_ + β_3_INT_it_・TIME_it_ + β_4_CTRL_it_ + β_5_TIME_it_・CTRL_it_ + β_6_AGE1_it_ + β_7_AGE2_it_ + β_8_SEX_it_ + ε_it_* where Y: outcome variable, INT: intervention to 20% coinsurance rate from 10% TIME: baseline per-month trend, CTRL: treatment/control groups indicator, AGE1: age group of 80–84 years, AGE2: age group of 85+ years, and SEX: sex

Abbreviations: RR, relative ratio; 95% CI, 95% confidence interval; CIR, coinsurance rate

**Table S3. Association between increase of coinsurance rate and outpatient care expenditure and utilization based on single arm interrupted time series analysis (n = 22,013).**

| Total expenditure:  Overall outpatient care | RR | 95% CI | | p-value |
| --- | --- | --- | --- | --- |
|  |  | LL | UL |  |
| Change due to the increase  in CIR to 20% | 0·93 | 0·92 | 0·95 | <0·001 |
| Trend before the increase in CIR to 20% | 1·005 | 1·003 | 1·007 | <0·001 |
| Slope change after the increase  in CIR to 20% | 1·002 | 0·999 | 1·005 | 0·172 |
| Total expenditure:  Physician visits | RR | 95% CI | | p-value |
|  |  | LL | UL |  |
| Change due to the increase  in CIR to 20% | 0·92 | 0·90 | 0·94 | <0·001 |
| Trend before the increase in CIR to 20% | 1·005 | 1·003 | 1·008 | <0·001 |
| Slope change after the increase  in CIR to 20% | 1·006 | 1·002 | 1·010 | 0·003 |
| Total expenditure:  Prescription | RR | 95% CI | | p-value |
|  |  | LL | UL |  |
| Change due to the increase  in CIR to 20% | 0·95 | 0·93 | 0·97 | <0·001 |
| Trend before the increase in CIR to 20% | 1·006 | 1·004 | 1·009 | <0·001 |
| Slope change after the increase  in CIR to 20% | 0·996 | 0·992 | 0·999 | 0·022 |
| Total expenditure:  Dental visits | RR | 95% CI | | p-value |
|  |  | LL | UL |  |
| Change due to the increase  in CIR to 20% | 0·97 | 0·93 | 1·01 | 0·125 |
| Trend before the increase in CIR to 20% | 0·998 | 0·994 | 1·002 | 0·398 |
| Slope change after the increase  in CIR to 20% | 0·996 | 0·990 | 1·002 | 0·224 |
| Out-of-pocket expenditure  Overall outpatient care | RR | 95% CI | | p-value |
|  |  | LL | UL |  |
| Change due to the increase  in CIR to 20% | 1·60 | 1·58 | 1·61 | <0·001 |
| Trend before the increase in CIR to 20% | 1·003 | 1·002 | 1·004 | <0·001 |
| Slope change after the increase  in CIR to 20% | 1·002 | 1·000 | 1·003 | 0·046 |

NOTE: The estimates were predicted using standardization based on the regression model as follows: *Y_it_ = α + β_1_INT_it_ + β_2_TIME_it_ + β_3_INT_it_・TIME_it_ + β_4_AGE1_it_ + β_5_AGE2_it_ + β_6_SEX_it_ + ε_it_* where Y: outcome variable, INT: intervention to 20% coinsurance rate from 10% TIME: baseline per-month trend, AGE1: age group of 80-84 years, AGE2: age group of 85+ years, and SEX: sex.

Abbreviations: RR, relative ratio; 95% CI, 95% confidence interval; LL, lower limit; UL, upper limit; CIR, coinsurance rate.

**Table S4. Association between the increase of coinsurance rate and outpatient care expenditure based on the two-part model (n = 126,474).**

| Total expenditure | First-part: Logistic regression model | | | |  | Second-part: Log-gamma regression model | | | |
| --- | --- | --- | --- | --- | --- | --- | --- | --- | --- |
| Overall outpatient care | OR | 95% CI | | p |  | RR | 95% CI | | p |
|  |  | LL | UL |  |  |  | LL | UL |  |
| Change due to the increase  in CIR to 20% | 0·87 | 0·85 | 0·89 | <0·001 |  | 0·96 | 0·94 | 0·97 | <0·001 |
| Trend before the increase in CIR to 20% | 1·008 | 1·005 | 1·010 | <0·001 |  | 1·004 | 1·002 | 1·005 | <0·001 |
| Slope change after the increase  in CIR to 20% | 0·992 | 0·989 | 0·996 | <0·001 |  | 1·003 | 1·001 | 1·006 | 0·015 |
| Physician visits | OR | 95% CI | | p |  | RR | 95% CI | | p |
|  |  | LL | UL |  |  |  | LL | UL |  |
| Change due to the increase  in CIR to 20% | 0·86 | 0·84 | 0·87 | <0·001 |  | 0·95 | 0·93 | 0·97 | <0·001 |
| Trend before the increase in CIR to 20% | 1·007 | 1·005 | 1·010 | <0·001 |  | 1·004 | 1·001 | 1·006 | 0·004 |
| Slope change after the increase  in CIR to 20% | 0·997 | 0·994 | 1·001 | 0·101 |  | 1·006 | 1·003 | 1·010 | 0·001 |
| Prescription | OR | 95% CI | | p |  | RR | 95% CI | | p |
|  |  | LL | UL |  |  |  | LL | UL |  |
| Change due to the increase  in CIR to 20% | 0·90 | 0·89 | 0·92 | <0·001 |  | 0·99 | 0·97 | 1·01 | 0·323 |
| Trend before the increase in CIR to 20% | 1·009 | 1·008 | 1·011 | <0·001 |  | 1·002 | 1·000 | 1·005 | 0·039 |
| Slope change after the increase  in CIR to 20% | 0·996 | 0·993 | 0·998 | 0·001 |  | 0·998 | 0·994 | 1·001 | 0·170 |
| Dental visits | OR | 95% CI | | p |  | RR | 95% CI | | p |
|  |  | LL | UL |  |  |  | LL | UL |  |
| Change due to the increase  in CIR to 20% | 0·99 | 0·96 | 1·01 | 0·299 |  | 0·98 | 0·95 | 1·01 | 0·154 |
| Trend before the increase in CIR to 20% | 0·999 | 0·996 | 1·001 | 0·360 |  | 1·000 | 0·997 | 1·003 | 0·970 |
| Slope change after the increase  in CIR to 20% | 0·997 | 0·993 | 1·001 | 0·097 |  | 0·998 | 0·994 | 1·003 | 0·448 |
| Out-of-pocket expenditure:  Overall outpatient care | OR | 95% CI | | p |  | RR | 95% CI | | p |
|  |  | LL | UL |  |  |  | LL | UL |  |
| Change due to the increase  in CIR to 20% | 0·87 | 0·85 | 0·89 | <0·001 |  | 1·63 | 1·62 | 1·65 | <0·001 |
| Trend before the increase in CIR to 20% | 1·008 | 1·005 | 1·010 | <0·001 |  | 1·002 | 1·001 | 1·003 | <0·001 |
| Slope change after the increase  in CIR to 20% | 0·992 | 0·989 | 0·996 | <0·001 |  | 1·003 | 1·001 | 1·005 | <0·001 |

NOTE: The estimates were predicted using standardization based on the regression model as follows: *Y_it_ = α + β_1_INT_it_ + β_2_TIME_it_ + β_3_INT_it_・TIME_it_ + β_4_CTRL_it_ + β_5_TIME_it_・CTRL_it_ + β_6_AGE1_it_ + β_7_AGE2_it_ + β_8_SEX_it_ + ε_it_* where Y: outcome variable, INT: intervention to 20% coinsurance rate from 10% TIME: baseline per-month trend, CTRL: treatment/control groups indicator, AGE1: age group of 80-84 years, AGE2: age group of 85+ years, and SEX: sex.

Abbreviations: RR, relative ratio; 95% CI, 95% confidence interval; LL, lower limit; UL, upper limit; CIR, coinsurance rate.

**Table S5. Price elasticity of outpatient care expenditure due to increase of coinsurance rate based on two-part model (n = 22,013).**

|  | Outpatients care expenditure | | | | | | | | | | | | | | |
| --- | --- | --- | --- | --- | --- | --- | --- | --- | --- | --- | --- | --- | --- | --- | --- |
|  | Overall outpatient care | | |  | Physician visits | | |  | Prescription | | |  | Dental visits | | |
|  | Mean (JPY) | 95% CI | |  | Mean (JPY) | 95% CI | |  | Mean (JPY) | 95% CI | |  | Mean (JPY) | 95% CI | |
|  |  | LL | UL |  |  | LL | UL |  |  | LL | UL |  |  | LL | UL |
| Total expenditure |  |  |  |  |  |  |  |  |  |  |  |  |  |  |  |
| Q_1_: Before increase in  CIR to 20% | 32,636 | 32,095 | 33,177 |  | 19,810 | 19,399 | 20,221 |  | 9,757 | 9,465 | 10,048 |  | 3,054 | 2,978 | 3,130 |
| Q_2_: After increase in  CIR to 20% | 30,493 | 29,985 | 31,001 |  | 18,279 | 17,879 | 18,680 |  | 9,250 | 8,997 | 9,504 |  | 2,959 | 2,885 | 3,033 |
| ∆Q = Q_2_ − Q_1_ | -2,143 | -2,620 | -1,666 |  | -1,530 | -1,935 | -1,126 |  | -506 | -707 | -306 |  | -94 | -199 | 11 |
|  |  |  |  |  |  |  |  |  |  |  |  |  |  |  |  |
| Out-of-pocket expenditure |  |  |  |  |  |  |  |  |  |  |  |  |  |  |  |
| P_1_: Before increase in  CIR to 20% | 3,023 | 2,991 | 3,055 |  | 1,827 | 1,802 | 1,851 |  | 893 | 879 | 908 |  | 302 | 295 | 309 |
| P_2_: After increase in  CIR to 20% | 4,821 | 4,763 | 4,879 |  | 2,847 | 2,801 | 2,893 |  | 1,481 | 1,451 | 1,512 |  | 492 | 481 | 503 |
| ∆P = P_2_ − P_1_ | 1,798 | 1,746 | 1,850 |  | 1,021 | 978 | 1,064 |  | 588 | 563 | 613 |  | 190 | 177 | 203 |
|  |  |  |  |  |  |  |  |  |  |  |  |  |  |  |  |
| Point price elasticity | -0·110 | - | |  | -0·138 | - | |  | -0·079 | - | |  | -0·049 | - | |
| Arc price elasticity | -0·148 | - | |  | -0·184 | - | |  | -0·108 | - | |  | -0·065 | - | |

*NOTE1*: The estimates were predicted using standardization based on the regression model as follows: *Y_it_ = α + β_1_INT_it_ + β_2_TIME_it_ + β_3_INT_it_・TIME_it_ + β_4_CTRL_it_ + β_5_TIME_it_・CTRL_it_ + β_6_AGE1_it_ + β_7_AGE2_it_ + β_8_SEX_it_ + ε_it_* where Y: outcome variable, INT: intervention to 20% coinsurance rate from 10% TIME: baseline per-month trend, CTRL: treatment/control groups indicator, AGE1: age group of 80-84 years, AGE2: age group of 85+ years, and SEX: sex.

*NOTE2*: Point price elasticity = $\frac{\Delta Q/Q_{1}}{\Delta P/P_{1}}$, Arc price elasticity = $\frac{P_{1}+P_{2}}{Q_{1}+Q_{2}} \times\frac{\Delta Q}{\Delta P}$

*Abbreviations*: 95% CI, 95% confidence interval; LL, lower limit; UL, upper limit; CIR, coinsurance rate.

**Table S6. Price elasticity of outpatient care expenditure due to increase of coinsurance rate based on donut hole model ^a^ (n = 22,013).**

|  | Outpatients care expenditure | | | | | | | | | | | | | | |
| --- | --- | --- | --- | --- | --- | --- | --- | --- | --- | --- | --- | --- | --- | --- | --- |
|  | Overall outpatient care | | |  | Physician visits | | |  | Prescription | | |  | Dental visits | | |
|  | Mean (JPY) | 95% CI | |  | Mean (JPY) | 95% CI | |  | Mean (JPY) | 95% CI | |  | Mean (JPY) | 95% CI | |
|  |  | LL | UL |  |  | LL | UL |  |  | LL | UL |  |  | LL | UL |
| Total expenditure |  |  |  |  |  |  |  |  |  |  |  |  |  |  |  |
| Q_1_: Before increase in  CIR to 20% | 32,160 | 31,565 | 32,756 |  | 19,779 | 19,292 | 20,267 |  | 9,319 | 9,039 | 9,600 |  | 3,116 | 3,013 | 3,219 |
| Q_2_: After increase in  CIR to 20% | 30,854 | 30,233 | 31,474 |  | 18,315 | 17,823 | 18,807 |  | 9,619 | 9,300 | 9,938 |  | 2,956 | 2,859 | 3,054 |
| ∆Q = Q_2_ − Q_1_ | -1,307 | -2,065 | -548 |  | -1,464 | -2,133 | -796 |  | 300 | -13 | 612 |  | -160 | -331 | 11 |
|  |  |  |  |  |  |  |  |  |  |  |  |  |  |  |  |
| Out-of-pocket expenditure |  |  |  |  |  |  |  |  |  |  |  |  |  |  |  |
| P_1_: Before increase in  CIR to 20% | 2,982 | 2,944 | 3,019 |  | 1,823 | 1,793 | 1,853 |  | 857 | 841 | 873 |  | 307 | 298 | 317 |
| P_2_: After increase in  CIR to 20% | 4,876 | 4,807 | 4,945 |  | 2,852 | 2,798 | 2,907 |  | 1,538 | 1,501 | 1,574 |  | 492 | 477 | 507 |
| ∆P = P_2_ − P_1_ | 1,894 | 1,821 | 1,967 |  | 1,030 | 969 | 1,090 |  | 681 | 645 | 717 |  | 184 | 163 | 205 |
|  |  |  |  |  |  |  |  |  |  |  |  |  |  |  |  |
| Point price elasticity | -0·064 | - | |  | -0·131 | - | |  | 0·040 | - | |  | -0·086 | - | |
| Arc price elasticity | -0·086 | - | |  | -0·175 | - | |  | 0·056 | - | |  | -0·114 | - | |

^a^ Donut hole model excluded the data before and after two months of intervention (08/01/2022 to 11/30/2022).

*NOTE1*: The estimates were predicted using standardization based on the regression model as follows: *Y_it_ = α + β_1_INT_it_ + β_2_TIME_it_ + β_3_INT_it_・TIME_it_ + β_4_CTRL_it_ + β_5_TIME_it_・CTRL_it_ + β_6_AGE1_it_ + β_7_AGE2_it_ + β_8_SEX_it_ + ε_it_* where Y: outcome variable, INT: intervention to 20% coinsurance rate from 10% TIME: baseline per-month trend, CTRL: treatment/control groups indicator, AGE1: age group of 80-84 years, AGE2: age group of 85+ years, and SEX: sex.

*NOTE2*: Point price elasticity = $\frac{\Delta Q/Q_{1}}{\Delta P/P_{1}}$, Arc price elasticity = $\frac{P_{1}+P_{2}}{Q_{1}+Q_{2}} \times\frac{\Delta Q}{\Delta P}$

*Abbreviations*: 95% CI, 95% confidence interval; LL, lower limit; UL, upper limit; CIR, coinsurance rate.

**Table S7. Price elasticity of outpatient care utilization due to increase of coinsurance rate (n = 22,013).**

|  | Physician visits | | |  | Prescription | | |  | Dental visits | | |
| --- | --- | --- | --- | --- | --- | --- | --- | --- | --- | --- | --- |
|  | Mean | 95% CI | |  | Mean | 95% CI | |  | Mean | 95% CI | |
|  |  | LL | UL |  |  | LL | UL |  |  | LL | UL |
| Visits/month (times) |  |  |  |  |  |  |  |  |  |  |  |
| Q_1_: Before increase to  20% coinsurance rate | 2.15 | 2.12 | 2.18 |  | 0.963 | 0.952 | 0.974 |  | 0.401 | 0.393 | 0.409 |
| Q_2_: After increase to  20% coinsurance rate | 1.96 | 1.93 | 1.99 |  | 0.881 | 0.871 | 0.892 |  | 0.397 | 0.388 | 0.405 |
| ∆Q = Q_2_ − Q_1_ | -0.19 | -0.21 | -0.17 |  | -0.081 | -0.090 | -0.073 |  | -0.004 | -0.015 | 0.007 |
|  |  |  |  |  |  |  |  |  |  |  |  |
| Out-of-pocket expenditure/month (JPY) |  |  |  |  |  |  |  |  |  |  |  |
| P_1_: Before increase to  20% coinsurance rate | 1,827 | 1,801 | 1,852 |  | 894 | 878 | 909 |  | 305 | 298 | 313 |
| (P_2_: After increase to  20% coinsurance rate | 2,849 | 2,802 | 2,896 |  | 1,482 | 1,451 | 1,513 |  | 496 | 485 | 508 |
| ∆P = P_2_ − P_1_ | 1,022 | 979 | 1,066 |  | 589 | 564 | 613 |  | 191 | 177 | 206 |
|  |  |  |  |  |  |  |  |  |  |  |  |
| Point price elasticity | -0.159 | - | |  | -0.128 | - | |  | -0.017 | - | |
| Arc price elasticity | -0.212 | - | |  | -0.178 | - | |  | -0.022 | - | |

*NOTE1*: The estimates were predicted using standardization based on the regression model as follows: *Y_it_ = α + β_1_INT_it_ + β_2_TIME_it_ + β_3_INT_it_・TIME_it_ + β_4_CTRL_it_ + β_5_TIME_it_・CTRL_it_ + β_6_AGE1_it_ + β_7_AGE2_it_ + β_8_SEX_it_ + ε_it_* where Y: outcome variable, INT: intervention to 20% coinsurance rate from 10% TIME: baseline per-month trend, CTRL: treatment/control groups indicator, AGE1: age group of 80-84 years, AGE2: age group of 85+ years, and SEX: sex.

*NOTE2*: Point price elasticity = $\frac{\Delta Q/Q_{1}}{\Delta P/P_{1}}$, Arc price elasticity = $\frac{P_{1}+P_{2}}{Q_{1}+Q_{2}} \times\frac{\Delta Q}{\Delta P}$

*Abbreviations*: 95% CI, 95% confidence interval; LL, lower limit; UL, upper limit; CIR, coinsurance rate.

**
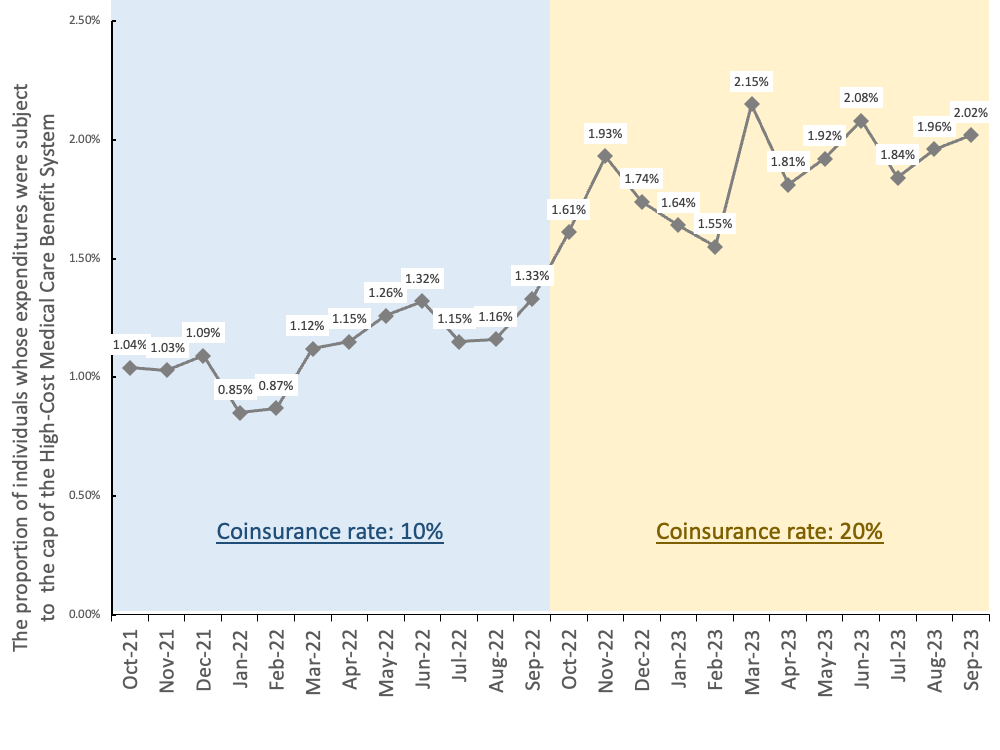
**

**Figure S4. The proportion of individuals whose expenditures were subject to the High-Cost Medical Care Benefit System (n = 22,013).**

NOTE: The estimate difference between before and after cost-sharing reform was 0.39% (95%CI: 0.27%, 0.51%). The estimates were predicted using standardization based on the regression model as follows: *Y_it_ = α + β_1_INT_it_ + β_2_TIME_it_ + β_3_INT_it_・TIME_it_ + β_4_CTRL_it_ + β_5_TIME_it_・CTRL_it_ + β_6_AGE1_it_ + β_7_AGE2_it_ + β_8_SEX_it_ + ε_it_* where Y: outcome variable, INT: intervention to 20% coinsurance rate from 10% TIME: baseline per-month trend, CTRL: treatment/control groups indicator, AGE1: age group of 80-84 years, AGE2: age group of 85+ years, and SEX: sex.
